# Newborn Screening for Lysosomal Storage Diseases: Methodologies, Screen Positive Rates, Normalization of Datasets, Second-Tier Tests, and Post-Analysis Tools

**DOI:** 10.3390/ijns4030023

**Published:** 2018-07-09

**Authors:** Michael H. Gelb

**Affiliations:** 1 Departments of Chemistry, University of Washington, Seattle, WA 98195, USA; gelb@uw.edu; 2 Departments of Biochemistry, University of Washington, Seattle, WA 98195, USA

**Keywords:** newborn screening, lysosomal storage diseases, cutoff values, tandem mass spectrometry, diagnosis, prognosis, dried blood spots, enzymatic activity assays

## Abstract

All of the worldwide newborn screening (NBS) for lysosomal storage diseases (LSDs) is done by measurement of lysosomal enzymatic activities in dried blood spots (DBS). Substrates used for these assays are discussed. While the positive predictive value (PPV) is the gold standard for evaluating medical tests, current PPVs for NBS of LSDs cannot be used as a performance metric due to statistical sampling errors and uncertainty in the onset of disease symptoms. Instead, we consider the rate of screen positives as the only currently reliable way to compare LSD NBS results across labs worldwide. It has been suggested that the expression of enzymatic activity data as multiple-of-the-mean is a way to normalize datasets obtained using different assay platforms, so that results can be compared, and universal cutoffs can be developed. We show that this is often not the case, and normalization is currently not feasible. We summarize the recent use of pattern matching statistical analysis together with measurement of an expanded group of enzymatic activities and biomarkers to greatly reduce the number of false positives for NBS of LSDs. We provide data to show that these post-enzymatic activity assay methods are more powerful than genotype analysis for the stratification of NBS for LSDs.

## 1. Introduction

Lysosomal storage diseases (LSDs) comprise a family of ~50 inborn errors resulting from a defect in lysosomal function, which is usually due to mutations in an enzyme involved in the breakdown of cell components that target to the lysosome. Newborn screening (NBS) for LSDs started in Taiwan for Pompe disease, which is especially frequent in the Asian population, and studies showed that early intervention with enzyme replacement therapy led to improved outcomes in infantile patients [1]. NBS for Krabbe disease in New York started at about the same time, with mandatory statewide screening initiated by parent advocacy [2]. A combination of parent advocacy and legislative processes in other states led to the expansion of NBS programs (Pompe, Krabbe, Gaucher, Fabry, mucopolysaccharidosis-I (MPS-I), MPS-II, and Niemann–Pick A/B, or a subset) in Illinois, Kentucky, Massachusetts, Minnesota, Missouri, New Jersey, New York, Ohio, Pennsylvania, and Tennessee. In 2016, Pompe and MPS-I diseases were added to the Recommended Uniform Newborn Screening Panel (RUSP) by the Advisory Committee on Heritable Disorders in Newborns and Children (part of Health Resources and Services Administration, HRSA, in the United States), and several states are preparing to add these conditions in the near future. Table 1 lists all of the worldwide locations where one or more LSDs are being screened live at the time of this writing.

## 2. Enzymatic Activity-Based NBS for LSDs

All of the labs engaged in LSD NBS carry out first-tier testing by measurement of lysosomal enzymatic activities in dried blood spots (DBS) on NBS cards using either tandem mass spectrometry (MS/MS) or fluorimetry (either digital microfluidics (DMF-F) or a standard plate reader). This is rooted in the pioneering work of the late Nestor Chamoles, who showed that many lysosomal enzymes are catalytically active in re-hydrated DBS (for example, [3]).

MS/MS-based quantification of glycosaminoglycan-derived fragments derived from treatment of DBS extracts with bacterial heparinases have been proposed for NBS of the MPS diseases [4,5]. This is based on the hypothesis that one or more glycosaminoglycan species will accumulate if a lysosomal enzyme involved in their breakdown is deficient. In one method, glycosaminoglycan-derived disaccharides from heparinase treatment are quantified by liquid chromatography-MS/MS (LC-MS/MS) [5]. In this analysis, each glycosaminoglycan polymer liberates many disaccharides (i.e., ~50 disaccharides coming from a ~100-mer). In the non-reducing end method [6,7], only the carbohydrate at the non-reducing end of the glycosaminoglycan (1 per long polymer) is quantified owing to its unique MS/MS signature compared with the fragments coming from the internal part of the polymer. In the former method, it may be more difficult to observe the accumulated glycosaminoglycan material in an LSD sample, because the polymer that accumulates in the lysosome and the relatively large amount of cell surface polymer contribute to the disaccharide abundance, and each polymer generates many internal disaccharides. In the non-reducing end method, each glycosaminoglycan polymer generates only one non-reducing end, and the lysosome will contain a large amount of polymer with a structurally identical non-reducing end should there be a block in a breakdown step due to a deficient enzyme (i.e., in an MPS sample). A problem with both glycosaminoglycan methods for NBS is that they require much more extensive sample processing compared to the enzyme activity measurements, and they also require LC-MS/MS runs that last several minutes per sample (which is too slow for NBS). Furthermore, an initial pilot study of the glycosaminoglycan disaccharide method showed a very high rate of false positives for the MPS diseases compared to the enzyme-assay method [5] (discussed in detail, [8]). Pilot studies of the non-reducing end method have not been published, but are expected to have a lower false positive rate (see above).

In the case of Krabbe disease, current data shows that the false positive rate would be lower if the biomarker psychosine was measured as a first-tier test compared to measurement of the activity of the relevant enzyme galactocerebrosidase (GALC) (Section 6.4 below). However, the measurement of psychosine in DBS is too difficult for a first-tier analysis, as it requires a top-end MS/MS instrument in terms of analyte detection sensitivity together with a liquid chromatography run of several minutes. It is thus much more reasonable to use the much higher throughput measurement of GALC activity for first-tier analysis followed by psychosine analysis on the small subset of initially screen-positive samples. Similar arguments apply for glucosylsphingosine as a biomarker of Gaucher disease (Section 6.4, below). Lysosphingomyelin is elevated in DBS from Niemann–Pick A/B (Section 6.5, below). However, the degree of elevation compared with levels in normal controls is modest [9], suggesting that a high false positive rate would occur if this biomarker was used for first-tier NBS. Lyso-Gb3 is a useful biomarker for Fabry disease (Section 6.6, below), but there is continued controversy about whether it is always elevated in the newborn period.

## 3. Substrates Used for Enzymatic Activity Assays

All of the substrates used to assay the enzymatic activities of lysosomal enzymes in DBS make use of unnatural substrates. There may be concern that the use of these substrates does not convey the true extent to which an enzyme mutation affects the activity on the natural substrate. The only known example of this is the fluorimetric assay for acid sphingomyelinase, where the use of the 4-methylumbelliferone substrate leads to a significant false negative problem [10,11]. This is because ~10% of Niemann–Pick A/B patients contain a single copy of the Q292K mutant, which is sufficient to nearly abolish the enzymatic activity of acid sphingomyelinase on the natural substrate sphingomyelin. However, this mutant retains full activity on the artificial 4-methylumbelliferyl-substrate. The Q292K mutant binds the natural substrate very weakly, but apparently the artificial fluorogenic substrate binds nearly as well as it does to the wild type enzyme such that enzymatic activity is not compromised [11]. The substrate used for MS/MS is nearly identical to the natural sphingomyelin substrate; the only difference is the length of the fatty acyl chain attached to the sphingosine backbone [12]. The MS/MS assay does not suffer from this false negative problem [12]. Caution is thus advised in the interpretation of Niemann–Pick A/B results obtained with the fluorimetric substrate. The MS/MS substrates for Gaucher and Krabbe diseases are also essentially the natural substrates, again the only difference is the length of the fatty acyl chains.

The fluorometric substrate that is typically used to assay *N*-acetyl-galactosamine-4-sulfatase (also known as arylsulfatase B) for analysis of MPS-VI is a simple sulfate ester to an aromatic phenol [13], whereas the MS/MS substrate contains a carbohydrate portion that is identical to that of the natural substrate [8]. It is likely that other sulfatases will act on the aromatic sulfates to a finite extent, thus contributing non-zero activity in DBS when the MPS-VI enzyme is null. It remains to be established whether residual activity measured in confirmed MPS-VI patient DBS or leukocytes is due to an incomplete loss of *N*-acetyl-galactosamine-4-sulfatase or the action of other enzymes on the aromatic sulfate ester substrate. This has important consequences for prognosis studies.

For enzymes relevant to MPS diseases, Hopwood et al. studied substrates containing polysaccharides that are derived directly from the long chain glycosaminoglycans, and reported that these display higher activities than the monosaccharides on the enzymes that degrade these polymers (relevant to all MPSs) (for example, [14]). This is due mainly to the lower *K_m_* values with the polysaccharide substrates; the *k_cat_* values on the monosaccharides are similar to those for the polysaccharides. It is conceivable that mutant glycosaminoglycan processing enzymes exist that show greatly diminished enzymatic activity on natural substrates, but not on the artificial substrates (similar to the problem noted above with Niemann–Pick A/B), but such mutants have not been reported to date. The synthesis or semi-synthesis of disaccharide or longer substrates is expected to be prohibitively expensive.

The sphingolipid hydrolyzing enzymes work together with an activator protein (saposins) under the natural setting with cellular membranes [15]. The current thinking is that the activator protein removes the sphingolipid from the plane of the membrane bilayer for binding to the active site of the hydrolase. In vitro, these sphingolipid hydrolases display high activity in the absence of activator proteins as long as detergent is present. Variants of LSDs caused by mutations to the activator protein are much rarer than mutations to the enzyme itself (for example, metachromatic leukodystrophy [16]). These LSD variants will be missed by in vitro enzymatic activity assays for NBS.

## 4. Screen Positive Rates and Positive Predictive Values

### 4.1. NBS Comparison Metrics and the Lack of Reliable Positive Predictive Values.

The gold standard for any test is the positive predictive value (PPV), which has a clear definition. The PPV is defined as the fraction of test positives that are true positives. In the case of LSD NBS, the problem comes in the definition of test and true positives. The number of test positives is usually taken as the number of first-tier NBS tests, which are called positive by the NBS lab. Typically, these are the number of newborns who display lysosomal enzymatic activity below a specific cutoff value. However, even this needs careful definition. For example, some NBS labs carry out a repeat first-tier analysis on samples close to the cutoff, and often some of the first-round positives are not taken forward as positives. Some labs incorporate second-tier analysis methods on all of the first-tier positive samples (i.e., DNA sequencing or biomarker quantification), thus reducing the number of cases carried forward. It is thus important to clearly define what constitutes a positive test when calculating the PPV, and indicate the collection of tests that was performed.

A bigger concern occurs with the number of true positives. Some NBS labs include in their true positive counts newborns who are screen positive in the first-tier test and who show mutations that are known to be associated with late-onset LSDs. Yet, these newborns are typically asymptomatic at the time of the analysis, and some of these patients may never develop clinically measurable disease. For example, there seems to be no consensus among experts in the field as to what fraction of potential late-onset Pompe patients (those with low enzymatic activity in the NBS and mutations found in other late onset patients) will develop Pompe disease, and the protocol recommended by the expert panel is to monitor these patients for early symptoms based on follow-up exams and start enzyme replacement therapy only if symptoms develop [17]. It is important to note that not all of the combinations of late-onset mutations reported in previous symptomatic patients will lead to measurable disease, because these mutations are typically partially penetrant, and each *combination* of mutations has to be considered as a separate case.

The most serious problem comes from LSDs being very rare; for example, MPS-VII has an incidence of <1 in 300,000 [18]. Although a PPV can always be calculated from a sample dataset, a useful estimate of the PPV can only be made when the number of newborns tested is large compared to the reciprocal of the disease frequency.

For example, in our recent pilot (unpublished) for MPS-VII, we found two test positives out of the first 20,000 newborns, and one of these was a clinically confirmed MPS-VII patient. Thus, the PPV is 0.5. Yet in the next 80,000 tests, we did not find a single true positive MPS-VII. The PPV would be poorly estimated as zero if this latter dataset was analyzed first. Thus, it is clear that at this stage of LSD NBS, quoted PPVs are not useful as a metric to judge the quality of a NBS test. For example, a comparison of NBS methods based on PPVs for NBS of MPS-I (frequency of ~1 in 80,000) based on datasets of <200,000 newborns [19] is not meaningful.

Perhaps the most important parameter considered by NBS labs is the false negative rate, as labs are very concerned about missing affected patients. However, estimates of false negative rates are lacking for the obvious reason that there is no reliable way to know of LSD-related illness in a large population who were screen negative for the condition. The only method available is the surveillance of patients by the medical community and tracking confirmed LSD patients back to their NBS result. This is likely to be far from a comprehensive estimate of false negatives.

The second most important NBS test metric is arguably the false positive rate. It is not possible at this time to obtain accurate false positive data because of the problem with potential late onset disease that was mentioned above. We are not aware of any test that has sufficiently powerful prognostic value for indicating whether a late-onset LSD will result in symptoms or when symptoms may begin. The only metric that can be reliably obtained at this point is the number of screen positives normalized to the number of newborns screened (the screen positive rate). NBS labs are very interested in this parameter, as it determines the workload of post-first-tier studies that need to be carried out by the NBS team or by the medical care system outside the domain of the NBS lab.

Note that the rate of screen positive samples (expressed as the number of screen positives normalized to the number of newborns tested, i.e., screen positives per 100,000 newborns) is reliably obtained from live NBS programs, from pilot studies using de-identified DBS, and from prospective pilot studies. PPVs can only be obtained from live NBS programs and prospective pilot studies, but as noted already, there are no LSD NBS datasets reported to date that give stable estimates of PPVs.

The rate of screen positives obviously depends on the cutoff value chosen by each NBS lab. Some labs use more conservative cutoffs than others. A full discussion of this factor is beyond the scope of this review, but a recent rigorous statistical analysis shows that it is possible to reliably compare the screen positive rates obtained from datasets in which different cutoff values were used (M. H. Gelb and B. H. Robinson, manuscript in preparation). The analysis is based on probability distribution functions, and takes into account assay imprecision. The latter can be measured by carrying out multiple independent assays using an identical DBS sample. Imprecision data is available from the Centers for Disease Control and Prevention as certification reports for quality control standards (https://www.cdc.gov/labstandards/nsqap_resources.html).

### 4.2. Normalization of Data Sets

For reasons describe above, we focus our discussion on the screen positive rate. It is obvious that the screen positive rate will depend on the cutoff chosen by each lab; the higher the cutoff, the higher the screen positive rate with other factors being equal. Some labs choose more conservative cutoff values than others, especially those just getting started with a new condition. One way to compare NBS studies across different assay platforms is to simply convert absolute enzymatic activity values to a percent of population mean activity values (which is sometimes called the multiple-of-the-mean) so that multiple assays can be equivalently scaled. For example, if the substrate used in one assay has a higher intrinsic activity with the lysosomal enzyme than the substrate used in a different assay, the mean activity across the population could be converted to 100% activity for both assays. If there are no other confounders, then it is reasonable to compare the number of screen positives for the two assay platforms at the equivalent cutoff of say 20% of mean activity.

Most mutations to lysosomal enzymes that lead to an LSD cause misfolding of the protein, and thus a lower amount of functional enzyme. In this case, the degree of enzymatic activity loss in terms of percent of activity of the non-mutated enzyme will almost certainly be independent of the structure of the substrate used in the enzymatic activity assay. It is possible that a point mutation could reduce the activity of one substrate more than for another (i.e., the Q292K mutant of acid sphingomyelinase discussed in Section 3), but these are thought to be exceptional cases. This again suggests that the use of percent of mean activity constitutes equivalent cutoff values for different assays.

A serious complication for the use of percent of mean activity as an equivalent cutoff comes when there is more than one enzyme that can give rise to the substrate-to-product conversion being measured in the assay. This is particularly relevant to Pompe disease and MPS-I. In the case of Pompe disease, it is well established that maltose glucoamylase hydrolyzes the same substrates used to assay the Pompe enzyme (GAA) and at the same pH values in the acidic range of near pH 4–5 [20]. This interference is significant, and it was not possible to carry out NBS for Pompe disease by measuring enzymatic activity in DBS until the reported use of acarbose as a selective inhibitor of maltose glucoamylase [20,21]. It is possible that for two different LSD enzymatic activity assays, there are different extents of inhibition of the interfering enzyme. This would significantly change the percent of mean activity for identical samples measured with the two different assays. This can easily be spotted in the case of Pompe disease by looking at the enzymatic activities in DBS from patients that have been confirmed as having infantile Pompe disease. These patients have virtually no residual GAA activity, and a significant amount of activity measured for these samples would suggest incomplete inhibition of the maltose glucoamylase. Table 2 gives the GAA enzymatic activity in DBS reported from patients that were confirmed to have Pompe disease in Missouri and New York. The mean GAA activity for infantile Pompe patients in Missouri and New York are 17.3% and 4.8% of population mean, respectively. It is well established that patients with infantile Pompe disease have virtually no residual GAA activity, and many are CRIM-negative [22], and the New York data is more in line with that expectation. The data suggest that the amount of acarbose used in the Missouri assay with DMF-F is insufficient to block all of the maltose glucoamylase in DBS. The data also show that it is invalid to compare the screen positive rates in Missouri and New York by using the same percent of mean GAA activity as an equivalent cutoff.

In the case of IDUA enzymatic activity for MPS-I, it is likely that all of the synthetic substrates used are contaminated with the beta-isomer of the iduronide-glycoside. This is because the IDUA substrates are made by chemical isomerization of the beta-isomeric synthetic intermediate, and isomerization is typically incomplete. Any beta-isomer would be hydrolyzed by beta-glucuronidase in the DBS, as the activity of this enzyme is even higher than that of IDUA. This would give rise to a false high activity of IDUA in samples deficient in IDUA. The solution has been to use d-saccharic acid-1,4-lactone as a beta-glucuronidase inhibitor. If the beta-glucuronidase in two different assays is fully inhibited by this agent, then one can presumably use the same percent of mean activity as an equivalent cutoff for both assays. Thus, one should carefully inspect the experimental conditions reported for each IDUA assay to assess whether a beta-glucuronidase inhibitor was used. In the early pilot study of MPS-I and other LSDs using MS/MS [24], the IDUA enzymatic activities were generally higher than those found in a latter study [25]. The difference is because the β-glucuronidase inhibitor was only used in the earlier study. Thus, it is not possible to compare these studies by using multiple-of-the-mean IDUA activities. On the other hand, it is valid to use the same percent of mean IDUA activity as an equivalent cutoff to compare screen positive rates in two pilot studies of MPS-I if β-glucuronidase is fully inhibited by the added inhibitor.

An additional example is the analysis of *N*-acetylgalactosamine-6-sulfatase for NBS of MPS-IVA with the fluorimetric assay with DBS. In one study using a 96-well plate reader, the mean activity measured for 25 confirmed MPS-IVA patients was 0.7% of the mean activity measured in 54 normal controls [26]. Meanwhile, another study using the same assay platform reported a mean activity for 13 MPS-IVA patients of 42% of the mean activity of 75 normal controls [27]. Normalization of these two datasets would be very difficult.

In conclusion, use of multiple-of-the-mean seems like a good approach to normalize NBS datasets obtained with different enzyme assay platforms, but extreme caution is needed in cases where there is measurable interference from off-target enzymes.

## 5. Pattern Recognition Statistical Tools Combined with Biomarker Analysis

Over the past decade, the group at the Mayo Clinic (Rochester, MN, USA) led by P. Rinaldo and colleagues have been carrying on post-analysis biostatistical tools to reduce the false positives in NBS (for example, [28]). Very recently, this group has applied these methods to NBS of LSDs [29]. The study is for the first year of mandated NBS of MPS-I, Krabbe, and Pompe in Kentucky (carried out by the Mayo clinic). Here, we give a brief summary of the approach and the results.

The post-analysis process involves multiple steps. (1) The enzymes relevant to MPS-I, Krabbe, and Pompe disease display a clear covariation with birth weight and gestational age, and thus each enzymatic activity is adjusted for these covariates using a statistically rigorous interpolation method. (2) A training set consisting of ~20 false positive DBS and ~20 true positive DBS for each of the three LSDs is submitted to a 6-plex enzymatic assay that includes the enzymes relevant to the three LDSs and also enzymes relevant to Gaucher, Fabry, and Niemann–Pick A/B using MS/MS [30]. This dataset is used together with a biostatistical pattern-matching tool called the CLIR Single Condition Tool. In the first step, all of the individual enzymatic activities and all of the ratios of activities are evaluated to find those that are “informative” (values for the true positives are noticeably separated from the true negatives). For example, for Krabbe disease, GALC activity alone and the activity ratios GALC/GBA, GALC/GLA, GALC/GAA, and GALC/ASM emerge as the only informative quantities [30]. In the prospective NBS phase (i.e., outside of the training set), each DBS is submitted to the 6-plex enzyme assay, and the tool assigns a score based on how many of the five informative quantities measured for each DBS lie within the range found in the true positives of the training set. For example, a score of zero is given to a new DBS measurement when all five informative quantities lie outside the range of values for the true positives. Likewise, a maximum score is given when all five informative quantities lie within the range for true positives. The beauty of this method is that intermediate scores can also be given when the DBS value falls between the two extreme patterns described above.

All DBS with a score >0 are moved forward into the next phase of the analysis. In this way, out of 55,161 newborns tested in Kentucky, 181 DBS survived for Krabbe, 76 survived for MPS-I, and 397 survived for Pompe. By using all of the scores of >0, a conservative approach is taken, and it seems very unlikely that a true positive would be left behind (i.e., false negative).

The next stage is based on the CLIR Dual Scatter Plot Tool. The *x*-axis of this plot is the true positive versus false positive score, which is maximal when the measured value hits all of the true positive informative values and misses all of the false positive values, as described above. The *y*-axis is the false positive versus true positive score, which is maximal for the opposite alignment (hits mainly false positive informative values). In this plot, true positives tend to appear in the lower right quadrant (high score for true positive and low score for false positive). True false positives appear in the upper right quadrant, and samples appearing in the off diagonal quadrants as taken as inconclusive. In this way, four samples are left for Krabbe, three are left for MPS-I, and five are left for Pompe.

The last stage is to carry out a repeat 6-plex enzymatic activity on the surviving samples and to also measure four lysophosphatidylcholine biomarkers (C20-C26 LPCs) (10-plex analysis) and carry out additional biomarker second-tier analysis as follows: (1) psychosine for Krabbe disease (Section 6.3, below); (2) glycosaminoglycan-derived disaccharides for MPS-I (Section 6.3 below), and the ratio of creatine to creatinine for Pompe (Section 6.1, below). As for the 6-plex, informative quantities are found by the CLIR tool, and for the 10-plex, and a score is given based on how well the sample aligns with the informative markers. If the 10-plex score is low and/or the second-tier test is negative, the sample is considered screen negative. After this stage, only a single Krabbe newborn out of 55,161 was left, and genotyping showed two severe mutations in trans. This patient received a neurological exam, and subsequently a bone marrow transplant. One MPS-I newborn was left, and genotyping showed two well-known pathogenic mutations in trans. This patient was evaluated and transplanted. Two Pompe newborns were left, and genotypes were consistent with the potential to develop late onset disease. These patients are being regularly monitored for signs of disease, but have not yet been placed on enzyme replacement therapy.

One concern about pattern matching/artificial intelligence-type post-analysis methods is that correlations between multiple variables can often be found in training sets, but these may not hold for future datasets. For example, for the past 30 years, every time baseball team X wins the first game of the season, they go on to the post-season playoffs. This statement, while easily verified, is unlikely to be predictive beyond the training set. Said another way, you can always find correlations in training sets if you consider enough variables. In contrast, the informative quantities for CLIR are fairly transparent. For example, the informative quantities for Krabbe are GALC activity and ratios of two lysosomal enzyme activities with GALC in the numerator (i.e., GALC/GAA, etc.). All are predicted to be low in a true Krabbe patient, but by using the ratios in addition to GALC, one is normalizing for DBS being an imperfect sampling method. For example, most of the lysosomal enzymes in blood are in leukocytes, and the use of a consistent volume of blood (by taking a 3-mm punch of a DBS) does not ensure that equal numbers of leukocytes are present in each NBS assay. The use of ratios of enzymatic activities is a logical way to mitigate against these variations if one assumes that multiple lysosomal enzymes vary by the same factor due to variation of the leukocyte number. Very long-chain lysophosphatidycholines (C20-26 LPC) used in the 10-plex CLIR method are presumably other normalization factors, since these lipids are present mainly in leukocytes rather than in plasma. Environmental factors and incomplete saturation of the paper by blood may lead to variation in enzymatic activity levels, but less so for the ratio of activities.

Surveillance over the coming years will likely address whether affected patients were missed by NBS with post-analysis CLIR tools (false negatives), but assuming a significant false negative problem does not exist; the way forward is clearly to combine enzymatic activity measurements in DBS with post-analysis CLIR biostatistical tools combined with second-tier biomarker quantification. The Mayo/KY study is by far the most precise LSD NBS program reported worldwide. It is interesting to note that although the KY NBS data is prospective, the same conclusions about the performance of the method, including CLIR post-analysis, would have been obtained in a pilot with de-identified samples. This is because the genotypes of the four patients who remained after the filtering are essentially conclusive, except in the case of Pompe disease, where the patients are asymptomatic and are suggestive to be true positives based only on a positive NBS test and genotyping. However, a prospective pilot study would not solve this Pompe-specific dilemma.

Some NBS labs have elected to test only for Pompe and MPS-I enzymatic activities, given that these are on the RUSP and in response to their mandate. Given the Mayo/KY results, consideration should be given to running a panel of more than two lysosomal enzymes. Although this will increase reagent costs, the cost savings due to a substantial drop in the number of false positives may be more than sufficient to justify the expanded reagent costs. Furthermore, the drop in the amount of family anxiety associated with false positives is an enormous benefit. New York is taking an intermediate approach of doing a 3-plex LSD panel (Krabbe, Pompe, and MPS-I for their mandate) and applying conventional cutoff methods for each disease. DBS that have a below-cutoff value are submitted to the 6-plex LSD panel followed by CLIR post-analysis. In this case, the increased reagent cost is offset by carrying out the 6-plex on only a tiny fraction of initial positives. New York is running this CLIR approach in parallel with their status quo approach (cutoffs only) in evaluation mode, and may soon opt to incorporate CLIR into their live NBS protocol based on encouraging results for the CLIR method (J. Orsini, APHL NBS Symposium, New Orleans, 2017). Wisconsin screens only for Pompe disease, performs the same 6-plex assay as KY, and uses the CLIR tools. CLIR tools can also be established when less than six enzymes are measured, but presumably, they will be less powerful.

## 6. NBS Strategies for Individual LSDs

### 6.1. General Remarks

Here, we give strategies that are emerging as the best way forward to analyze newborns who are deemed to be at high risk of developing an LSD based on nominally low lysosomal enzymatic activities in the initial DBS screen. We focus on those LSDs for which an FDA-approved treatment is available, or a non-approved treatment is in widespread use.

### 6.2. Pompe Disease

The only first-tier NBS method for Pompe disease is the measurement of GAA enzymatic activity in DBS in the presence of acarbose to inhibit maltose glucoamylase (Section 4.2). A very recent study shows that the ratio of [creatine/creatinine]/(GAA enzymatic activity) emerges as a useful post-analysis method for reducing false positives for Pompe disease [31]. This ratio is likely to be an indicator of muscle pathology, and is thus relevant to Pompe disease and likely other skeletal muscle diseases. Additional data is needed to gain confidence with this method, but initial results are encouraging. The measurement of glucose tetrasaccharide in urine (for example, [32]) may be useful, but this marker remains highly controversial because of its lack of accuracy for predicting Pompe disease.

GAA genotyping is typically done by some NBS labs, and two severe mutations in trans is likely to result in infantile-onset Pompe disease. However, a combination of late-onset mutations does not guarantee the development of Pompe disease later in life. The very large NY NBS program has recently uncovered a large number of GAA variants that are difficult to interpret (Colleen Stevens, unpublished data presented at the APHL NBS conference, New Orleans, 2017). In short, genotyping for Pompe disease has value in some cases, but remains highly problematic.

Recently, it has been shown that very precise measurements of residual GAA enzymatic activity in leukocytes by LC-MS/MS can separate infantile-onset from potential late-onset Pompe newborns [33]. The ability to measure tiny amounts of residual activity (i.e., <1% residual GAA activity in 0.2% increments) that are statistically significant has been proven by cell mixing studies using lymphoblasts from a GAA-null patient and cells from a GAA normal patient [33]. Time will tell how useful this LC-MS/MS analysis of GAA enzymatic activity is in leukocytes, but initial data look encouraging. It is generally accepted in the field that the fluorimetric assay of GAA with the 4-methylumbelliferone substrate have shown no difference in GAA enzymatic activities among early and late-onset Pompe patients.

As an aside, we note that the hypothesis that Pompe patients have lower leukocyte GAA enzymatic activities compared with those carrying pseudodeficiency mutations is strongly supported by recent studies with DBS [34]. Studies were carried out with DBS from newborns with two Pompe pathogenic mutations and those with pseudodeficiency mutations. MS/MS assay of GAA in DBS showed a clear separation between the two groups, whereas there was no separation seen in side-by-side studies with the fluorimetric GAA assay using the 4MU substrate [34]. Although a high precision analysis of GAA in leukocytes is preferred over DBS for second-tier analyses, the recent DBS data proves that variation in enzymatic activity due to DBS sampling is not overwhelming.

### 6.3. MPS-I

First-tier NBS is done worldwide by MS/MS or fluorimetric assay of α-iduronidase in DBS. The best option for second-tier analysis of screen positive samples appears to be analysis of glycosaminoglycan-derived olefinic disaccharides derived from the treatment of DBS with bacterial heparinases [4,5,35]. As described in Section 2, the non-reducing end glycosaminoglycan method is expected to give lower number of false positives, but the internal disaccharides methods in use are presumably sufficient in cases where α-iduronidase activity is low in the first-tier NBS analysis. Many NBS labs rely on genotyping for second-tier analysis, and as always for LSDs, poor understanding of potential late-onset DNA alterations and variations of unknown significance continue to be a problem, especially for prognosis. Although datasets of glycosaminoglycans in newborn DBS are not large, we are not aware of any exceptions to the observation of elevated levels in confirmed MPS-I patients (personal communication with S. Tomatsu, University of Delaware). The data seems clear that glycosaminoglycan analysis is more powerful than genotyping for second-tier stratification of screen positive samples.

### 6.4. Krabbe Disease

All first-tier NBS for Krabbe disease is based on measurement of the galactocerebrosidase (GALC) enzymatic activity in DBS using MS/MS [1,36] or fluorimetry with a 96-well plate reader. Pseudodeficiencies are commonly found with these methods, and genotyping as a second-tier method provides a clear indication of Krabbe disease only when two severe mutations are seen in trans [2]. Genotypes consistent with possible late onset Krabbe disease and variations of unknown significance (VOUS) continue to be a problem for diagnosis and prognosis [2].

One of the substrates of GALC is psychosine, and this lipid accumulates in DBS of Krabbe patients. Psychosine analysis by LC-MS/MS appears to be the most useful second-tier test reported to date [35,37,38]. All of the recently identified infantile Krabbe disease patients display elevated psychosine in DBS above ~10 nM [35,37,38]. In the New York Krabbe NBS program, leukocytes isolated from whole blood are obtained from most screen positive newborns for measurement of GALC activity using a radiometric assay [37]. Those patients with the lowest bracket of leukocyte GALC activity are placed into the high-risk category [2]. Over the past ~10 years of NBS for Krabbe disease in New York, ~20 high risk patients have been identified, of which five were confirmed to have infantile Krabbe disease, and the rest are so far asymptomatic (although some have been lost to follow-up) [2]. The original study reported elevated psychosine in the five infantile patients, but not in the asymptomatic high risk patients [38]. However, subsequent studies using an LC-MS/MS method with a lower limit of quantification [36] showed some variation in psychosine concentration in the asymptomatic high risk group, with some patients showing psychosine in the normal range of ~0.5–1.0 nM, with others showing up to a 10-fold increase in psychosine of ~5 nM. Studies are underway to measure psychosine in DBS in patients who have been confirmed to have late-onset Krabbe disease with the hope of generating a reference range for a form of the disease that is less severe than the infantile form (Gelb, M. H., Matern, D., Orsini, J. J., Escolar, M. L., unpublished).

The radiometric GALC assay in leukocytes used in New York does not distinguish infantile Krabbe patients from asymptomatic high risk newborns, but a recently developed LC-MS/MS assay of leukocyte GALC does [36]. The increased diagnostic power of the LC-MS/MS assay is probably related to its enormous resolution of small amounts of residual GALC (as is the case for Pompe disease, see Section 6.2). It was proven that increments of residual GALC of 0.2% or normal in the 0–1% of normal range can be detected [36]. Leukocyte GALC activity measured by LC-MS/MS from infantile Krabbe patients is virtually zero (<0.2%), whereas asymptomatic high risk patients display slightly higher residual activity of >~1% [36]. Although further studies are needed with an expanded set of patients, these initial studies suggest that the severity of Krabbe disease does correlate with the level of residual GALC activity in leukocytes, and that trace amounts of residual GALC activity prevent the development of the infantile phenotype.

In summary, psychosine in DBS and GALC activity in leukocytes both measured by LC-MS/MS are second-tier tests that seem to be more powerful than genotyping and radiometric assay of leukocyte GALC, and it is hoped that an increased use of these tests may be helpful for prognosis, so that follow-up of non-infantile high-risk patients can be optimized.

### 6.5. Gaucher Disease

NBS for Gaucher disease can be done by first-tier measurement of glucocerebrosidase in DBS by MS/MS or fluorescence. Glucosylsphingosine, which is measured by LC-MS/MS in DBS, has recently emerged as a very useful second-tier biomarker for Gaucher disease [39,40,41]. Care is required to selectively detect this biomarker, since it is isobaric with psychosine, and both lipids run close together during LC.

### 6.6. Niemann–Pick A/B

NBS for Niemann–Pick A/B disease can be done by first-tier measurement of acid sphingomyelinase in DBS by MS/MS or fluorescence. The use of the fluorimetric assay comes with the caution of the false negative problem, as described in Section 3. So far, only a few states carry out live NBS for Niemann–Pick A/B disease (Table 1), and this is done by LC-MS/MS assay of acid sphingomyelinase activity in DBS. Recent studies show that lysosphingomyelin is elevated in DBS from Niemann–Pick A/B patients [9], suggesting that LC-MS/MS analysis of this lipid could be a useful second-tier analysis prior to genotyping or other tests.

### 6.7. Fabry Disease

Worldwide NBS for Fabry disease is based on the measurement of α-galactosidase A activity in DBS by MS/MS or fluorimetry. Interference from the B isoform is minimized by the addition of high concentrations of *N*-acetylgalactosamine to the assay buffer [42]. There are several reports showing the elevation of the Fabry-relevant biomarker lyso-Gb3 and its analogs in Fabry patients (for example, [43,44,45,46,47]), but it is not clear if this biomarker is always elevated in newborn DBS from Fabry patients. NBS for Fabry disease remains very challenging given the large number of potential late-onset patients being discovered in programs that are now live for this LSD. Genotype-phenotype correlations, such as for other LSDs, are incompletely understood. A detailed analysis of the challenges of NBS for Fabry disease is beyond the scope of this review, and the best way forward is far from settled.

### 6.8. MPS-II, -IIIB, -IVA, -VI, and VII

The Chinese Foundation of Health (covering about 1/3 of newborns in Taiwan) is live for MS/MS-based NBS of MPS-II, -IVA, and -VI, and other NBS labs in Taiwan are planning to start NBS soon (Table 1). Data on the first ~120,000 newborns will be published shortly. We are conducting a MS/MS pilot study of NBS of these MPSs and MPS-VII in the WA NBS lab, and data collected so far for 80,000 newborns show an acceptable rate of false positives, suggesting that NBS for these disorders is feasible. A DMF-F assay for MPS-II has been reported [48], but no pilot studies have been reported. Fluorimetric assays for the enzymes relevant to MPS-IVA and –VI using a standard 96-well plate reader have been reported [13,26,27]. Some sulfatases, especially the enzymes relevant to MPS-IVA and MPS-VI are relatively slow enzymes, and thus an extended incubation time will be needed. For example, the plate-reader fluorimetric assay for the MPS-IVA enzyme is reported using 24 h or 48 h incubation times prior to reading the samples with the plate reader [26,27], and for DMF-F, the incubations are carried out in the same instrument that reads the fluorescence.

The MS/MS assay for MPS-IVA uses an *N*-acetylgalactosamine-6-sulfate-based substrate, which was shown to be a much faster substrate than those based on galactose-6-sulfate, such that incubation of 8–12 h yields an adequate signal [49,50]. This approach has been used to develop a new fluorimetric assay for MPS-IVA based on the 4MU-glycoside with *N*-acetyl-galactosamine-6-sulfate and an overnight incubation period (far shorter than the assay with the 4MU glycoside of galactose-6-sulfate described above). The buffer contains an inhibitor of hexosaminidase, since this enzyme liberates 4MU from the MPS-IVA substrate. The assay also contains a bacterial hexosaminidase that liberates 4MU only from the 4MU glycoside of *N*-acetylgalactosamine, which is the product of the MPS-IVA enzyme [49].

Second-tier testing for these additional MPSs will likely involve glycosaminoglycan disaccharide, as described in Section 2. Current data suggests that this analysis is more powerful than genotyping for disease diagnosis and prognosis. As far we as are aware from the literature data, glycosaminoglycans are always well elevated in confirmed MPS patients, but there is limited data during the newborn period.

### 6.9. Metachromatic Leukodystrophy

NBS for metachromatic leukodystrophy (MLD) by measurement of the arylsulfatase A activity in DBS is almost certainly not feasible because of the enormous pseudodeficiency problem [51]. Also, the enzyme is very unstable in DBS [52] (confirmed in our lab).

Recent studies show that the quantification of elevated sulfatides in DBS is the way forward for NBS [51]. We are conducting a pilot study in the WA NBS lab based on this approach, and have reached ~70,000 DBS, with only four false positives detected. A second approach for MLD NBS may be immunoquantification of arylsulfatase A in DBS, but reagents for this method have been discontinued (communications with D. Matern, Mayo Clinic, and J. Hopwood, Lysosomal Disease Research Unit, South Australia). Interest in MLD NBS is escalating because of the possibility of pre-symptomatic treatment with hematopoietic stem cell transplant especially when augmented with gene addition [53,54].

Second-tier testing should involve the measurement of sulfatides in urine by LC-MS/MS, as these are typically elevated to a higher level than in plasma [51].

### 6.10. Ceroid Lipofuscinosis II

This LSD is caused by deficiency of tripeptide peptidase-1 (TPP1). An MS/MS assay for TPP1 has been incorporated into the MPS LC-MS/MS multiplex [8], and we expect to add TPP1 to our ongoing MPS pilot study in 2018. The natural substrates for TPP1 are unknown, and there are no second-tier tests reported for this LSD. A fluorimetric assay of TPP-1 would also be possible.

### 6.11. Lysosomal Acid Lipase

Enzyme replacement therapy for lysosomal acid lipase (LAL) deficiency has recently been FDA-approved. Hamilton developed a useful assay for the detection of LAL in DBS using the fluorogenic lipase/esterase substrate palmitoyl-4MU [55]. This substrate is not specific for LAL. The LAL-contribution to the total esterase activity is obtained by two assays done in parallel, one with and one without the LAL-specific inactivator Lalistat-2. This assay is useful for the diagnosis of LAL deficiency. It is probably not sufficient for LAL NBS, because two assays are required per newborn, and the LAL activity is obtained as the difference between two nearly equal activity values. Thus, the residual will have a high error. Recently, we have discovered an analog of palmitoyl-4MU that is completely specific for LAL, and allows for its direct assay in DBS by MS/MS or fluorimetry [56]. As far as we are aware, there are no pilot or live NBS programs for LAL deficiency.

## Figures and Tables

**Table 1:** Newborn Screening (NBS) Programs Now Live for Lysosomal Storage Diseases (LSDs).^1^ MPS: Mucopolysaccharidosis.

Newborn Screening Program	LSDs Now Screened Live	NBS First-Tier Method
Illinois	Pompe, MPS-I, Gaucher, Fabry, Niemann–PickA/B, Krabbe, MPS-II	MS/MS enzymatic activity assay
Kentucky	Pompe, MPS-I, Krabbe	MS/MS enzymatic activity assay
Massachusetts	Pompe, MPS-I	MS/MS enzymatic activity assay
Minnesota	Pompe, MPS-I	MS/MS enzymatic activity assay
Mississippi	Pompe	MS/MS enzymatic activity assay
Missouri	Pompe, MPS-I, Gaucher, Fabry, Krabbe, MPS-II	DMF-F enzymatic activity assay (plate reader fluorescence assay for Krabbe)
New York	Pompe, Krabbe, MPS-I	MS/MS enzymatic activity assay
Ohio	Pompe, MPS-I, Krabbe	MS/MS enzymatic activity assay
Pennsylvania	Pompe, MPS-I, Krabbe, Fabry, Gaucher, Niemann Pick-A/B	MS/MS enzymatic activity assay
Wisconsin	Pompe	MS/MS enzymatic activity assay
Italy (Tuscany region)	Pompe, MPS-I, Fabry	MS/MS enzymatic activity assay
Italy (Veneto region)	Pompe, MPS-I, Fabry, Gaucher	MS/MS enzymatic activity assay
Taiwan (National Taiwan University Hospital, about 1/3 of newborns)	Pompe, MPS-I, Gaucher, Fabry, MPS-II, MPS-IIIB, MPS-IVA, MPS-VI	MS/MS and plate fluorimetry enzymatic activity assay
Taiwan (Chinese Foundation of Health, about 1/3 of newborns)	Pompe, MPS-I, Gaucher, Fabry, MPS-II, MPS-IIIB, MPS-IVA, MPS-VI	MS/MS enzymatic activity assay
Taiwan (Tapei Institute of Pathology, about 1/3 of newborns)	Pompe, MPS-I, Fabry, Gaucher, MPS-II	MS/MS enzymatic activity assay

^1^ Some states have mandatory NBS for LSDs and some have optional NBS.

**Table 2:** Data for Pompe Disease in the Missouri and New York NBS Labs.^1^

NBS Lab	Mean GAA Activity	GAA Activity for DBS from Confirmed Pompe Patients (μmol/h/L)	Cutoff	Mean GAA Activity for Infantile-Onset Pompe Disease
Missouri	27 μmol/h/L	Early-onset: 4.2, 4.4, 5.4, 5.1, 5.6, 5.2, 2.5, 4.6, 5.2 μmol/h/L	6.5 μmol/h/L	4.7 μmol/h/L
		Late-onset: 6.5, 5.2, 6.8, 6.7, 6.4, 5.8, 3.1, 5.5, 3.1, 5.5, 4.5, 5.7, 3.7, 5.4, 4.8, 4.1, 2.4, 3.4, 4.4, 2.5, 4.2, 4.9, 3.1 μmol/h/L	(24.1% of mean GAA)	(17.3% of mean GAA)
New York	10.6	Early-onset: 0.63, 0.69, 0.78, 0.22, 0.35, 0.41, 0.50 μmol/h/L	1.6 μmol/h/L	0.51 μmol/h/L
		Late-onset: 1.08, 0.89, 1.55, 1.69, 0.64, 1.19, 0.98, 0.70, 0.75, 0.99, 1.02, 1.21, 0.66, 0.37, 0.98, 0.58, 0.82, 0.63, 0.59, 0.88, 0.83, 1.43 μmol/h/L	(15% of mean GAA)	(4.8% of mean GAA)

^1^ Data from Missouri [23], data from New York (J. Orsini, Wadsworth Center).
